# Stability of asymmetric Schwarzschild–Rindler–de Sitter thin shell wormhole

**DOI:** 10.1038/s41598-024-63342-y

**Published:** 2024-06-03

**Authors:** A. Eid, A. Alkaoud, M. M. Khader, M. A. Bakry

**Affiliations:** 1 Department of Physics, College of Science, Imam Mohammad Ibn Saud Islamic University (IMSIU), Riyadh, Kingdom of Saudi Arabia; 2 Department of Astronomy, Faculty of Science, Cairo University, Giza, Egypt; 3 Department of Mathematics and Statistics, College of Science, Imam Mohammad Ibn Saud Islamic University (IMSIU), 11566 Riyadh, Saudi Arabia; 4 Department of Mathematics, Faculty of Science, Benha University, Benha, Egypt; 5 Department of Mathematics, Faculty of Education, Ain Shams University, Cairo, Egypt

**Keywords:** Astrophysics, Asymmetry thin shell wormhole, Exotic matter, Stability, Astronomy and planetary science, Physics

## Abstract

The paper examines the dynamics of asymmetric thin shell wormholes that connect two distinct spacetimes using the cut and paste technique. The focus is on analyzing the linear stability of these wormholes by considering radial perturbations and utilizing the modified generalized Chaplygin gas equation of state. The specific case of an asymmetric wormhole connecting Schwarzschild–Rindler spacetime to Schwarzschild–Rindler–de Sitter space–time is analyzed using this formalism. Our investigation uncovers the existence of both stable and unstable regions, which are contingent upon the appropriate selection of various parameters within the metric spacetime and equation of state. Additionally, we determine that stability regions exist as a consequence of the square speed of sound. By increasing the value of the cosmological constant, the stability region is expanded. Furthermore, the stability regions are augmented by the influence of Rindler parameters, while the stability regions are also affected by adjustments in the equation of state parameters, leading to their enlargement.

## Introduction

An asymmetric Schwarzschild–Rindler–de Sitter (SRdS) thin shell wormhole (TSW) is a theoretical geometric structure in general relativity that proposes the existence of a special type of wormhole composed of a thin, asymmetric shell mediating between two regions of four-dimensional spacetime with different characteristics. These theoretical structures have been studied by various researchers in the field of general relativity and wormholes, and theoretical analyses and complex calculations have shown that this type of wormhole could be theoretically possible according to Einstein’s equations^1,2^. The asymmetric thin-shell wormhole differs from other wormholes in its presence in a varying environment, where it exists in spacetime influenced by different forces such as gravity and cosmic expansion. This type of wormhole has been proposed for theoretical purposes and has not been extensively studied from a practical or experimental standpoint. The theoretical studies of asymmetric TSWs are part of ongoing research into understanding spacetime geometry and enigmatic phenomena like wormholes. By studying these theoretical structures, we can deepen our understanding of the nature of the universe and non-equilibrium fields in general relativity.

In 1966, a set formalism of invariant junction conditions at the surface of discontinuity was introduced by Israel^3^. There are two types of surfaces, such as boundary surfaces and surface layers. They play an important role in gravitational theory, both Newtonian and relativistic, and also in electromagnetic theory. Afterward, Visser^4^ investigated the idea of TSWs connecting two regions of different spacetimes or the same spacetimes by using cut and paste approach. These two regions are related by Israel junction conditions. Afterward, several authors discussed the construction and stability of TSWs in different theories with different equations of state (EoS). For instance, Poisson and Visser^5^ studied TSWs of two identical copies of Schwarzschild spacetimes. Gerica et al*.*^6^ studied the generic TSWs in relativity. The stability of various types of TSWs with different EoS has been extensively investigated by several researchers. Eiroa^7^ examined TSWs supported by a generalized Chaplygin gas (GCG) and studied their stability. Mazharimousavi et al*.*^8^ analyzed the stability of cylindrical TSWs. In another work, Mazharimousavi et al*.*^9^ analyzed the stability of TSW with a variable EoS. Varela^10^ investigated the stability of Schwarzschild TSWs with a variable EoS. Eid^11^ discussed the stability of cylindrical TSWs with Phantom energy. Eiroa^12^ conducted a stability analysis of TSWs in general. Kokubu and Harada^13^ discussed TSWs in the context of the Einstein-Gauss-Bonnet theory of gravity. Eid^1^ explored TSW stability in f(R) theory. Rahaman et al*.*^14^ studied the stability of TSWs in the context of Heterotic string theory. Finally, Eid^15^ investigated TSW stability in the Einstein-Hoffman-Born-Infeld theory. Recent studies have focused on analyzing the stability of asymmetric TSWs that exhibit different manifolds in the two regions of the throat. Forghani et al*.*^16^ investigated the stability of such asymmetric TSWs and addressed the issue of discontinuity in linear stability. They discussed the challenges associated with maintaining stability in these configurations. Additionally, Forghani et al*.*^17^ further explored the discontinuity in the linear stability of TSWs. In a related study, Eid^2^ examined asymmetric TSW stability with a variable EoS, shedding light on its behavior under different conditions.

In a series of papers Javed et al.^18–23^ have discussed thin shells in different physical contexts and different modified theories, e.g., the effect of a scalar field on the dynamical evolution of thin shell with hairy Schwarzschild black hole, the quantum corrected charged black hole solution bounded by quintessence, thin shell dynamics in regular charged black hole through T-duality, the stability of nonlinear electrodynamics thin shell wormholes via variable equations of state, new generic wormhole models with stability analysis via thin shell, stability analysis of new thin shell wormhole in teleparallel gravity. Also, Mustafa and coworkers^24,25^ have discussed the stability of thin shell wormholes within string cloud and quintessential field via the van der Waals and polytropic EOS, and also the construction of thin-shell around new wormhole solutions via solitonic quantum wave dark matter. In addition, Javed^26^ studied the geometric structure of a thin shell within the context of metric-affine gravity. Also, he studied the stability and dynamics of scalar field thin-shell for renormalization group improved Schwarzschild black holes^27^. Waseem et al.^28^ discussed the stability constraints of d-dimensional charged thin-shell wormholes via quintessence and a cloud of strings. Sharif and Javed^29^ studied the stability of charged thin-shell gravastars with quintessence. Moreover, Godani^30^ studied the linear and nonlinear stability of charged thin-shell wormholes in f(R) gravity. Mishra et al*.*^31^ studied the traversable wormhole models in f(R) gravity. Mazharimousavi^32^ discussed the thin shell wormhole satisfying the null-energy condition unconditionally. Hernández-Almada et al.^33^ discussed the cosmological constraints on the alternative model to Chaplygin fluid revisited. Debnath et al.^34^ analyzed the modified cosmic chaplygin AdS black hole. Bambi and Stojkovic^35^ discussed the astrophysical wormholes. De Celis and Simeone^36^ studied the traversability of thin-shell wormholes.

Recently, Grumiller^37^ investigated a new kind of model for the gravity of a central object at large scales. He obtained an extra new term that generates acceleration, similar to the ones observed in various abnormal systems in nature, called Rindler acceleration. Carloni et al*.*^38^ conducted a study examining the constraints imposed by the solar system on Rindler acceleration within the framework of general relativity. They investigated how Rindler acceleration, a concept related to the acceleration experienced by an observer in flat spacetime, affects the behavior of gravitational systems in our solar system. In a separate study, Sakalli and Mirekhtiary^39^ explored the influence of Rindler acceleration effects on Grumiller black hole (GBH) spectroscopy. They examined how the presence of Rindler acceleration, which is associated with non-inertial frames of reference, impacts the spectroscopic features of GBHs. This research sheds light on the connection between Rindler acceleration and the observable characteristics of black holes within the Grumiller model. Moreover, Sakalli and Ovgun^40^ studied the deflection of light and Hawking radiation from a Rindler- modified Schwarzschild BH. Afterward, Alestas et al*.*^41^ analyzed the stability of Schwarzschild–Rindler-anti de Sitter TSWs. Also, Muniz et al*.*^42^ studied the scalar particles around a Rindler-Schwarzschild wormhole.

Motivated by the aforementioned considerations, in the present our work is to investigate the dynamical and stability analysis of asymmetric TSW within the background geometry of Schwarzschild- Rindler- de Sitter spacetime under linear radial perturbation around the static solution via the modified generalized Chaplygin gas (MGCG) EoS. The paper is constructed as follows. In “Stability analysis of asymmetric Schwarzschild–Rindler–de Sitter TSW” section we display the dynamics and the stability analysis of asymmetric TSW under linear perturbation supported by MGCG EoS. Finally, a remarking conclusion is discussed in “Conclusion” section.

## Stability analysis of asymmetric Schwarzschild–Rindler–de Sitter TSW

Grumiller^37^ introduced an effective model called Rindler-modified Schwarzschild black hole geometry. He found a new term that generates acceleration (called Rindler acceleration) and leads to an abnormal acceleration in the geodesic of the test particle. The action describes the generic effective theory given by Grumiller,

1 $$S = - \int \left( { - R\Phi^{2} + 2\partial \Phi \partial \Phi + 8b\Phi - 6\Lambda \Phi^{2} + 2} \right)\sqrt { - g} d^{4} x,$$

where $g=det\left({g}_{\mu \nu }\right),$ $\Phi ,$ and $\Lambda$ are the determinants of the metric tensor, the scalar (dilaton) field, and the cosmological constant, with $R$ being the Ricci scalar curvature and $b$ representing the Rindler acceleration parameter.

Due to studying the influence of cosmological constant and Rindler acceleration on the stability of TSWs, regarding the background of static spherically symmetric line elements whose interior (−) and exterior (+)Schwarzschild–Rindler–de Sitter (SRdS) line elements are described by

2 $$ds_{ \pm }^{2} = - G_{ \pm } dt_{ \pm }^{2} + G_{ \pm }^{ - 1} dr_{ \pm }^{2} + h_{ \pm } \left( {d\theta_{ \pm }^{2} + \sin^{2} \theta_{ \pm } d\varphi_{ \pm }^{2} } \right),$$

and

3 $$G_{ \pm } \left( {r_{ \pm } } \right) = 1 - \frac{{2m_{ \pm } }}{{r_{ \pm } }} + 2 b_{ \pm } r_{ \pm } - \frac{1}{3} {\Lambda }_{ \pm } r_{ \pm }^{2} \quad {\text{and}}\quad h_{ \pm } \left( {r_{ \pm } } \right) = r_{ \pm }^{2} .$$

In the given context, the variables $m_{ \pm }$, ${b}_{\pm }$ and ${\Lambda }_{\pm }$ represent the mass of the central black hole object, the Rindler acceleration parameter, and the cosmological constant associated with both sides, respectively. When b equals zero, the solution of Schwarzschild-de Sitter is recovered. In the case where b and Λ are both zero, the solution of the Schwarzschild black hole is recovered. Furthermore, the metric (3) reduces to the two-dimensional Rindler metric, when m and Λ are equal to zero.

The stability of a static solution of radius ${r}_{^\circ }$ considering $\text{G}({r}_{^\circ })>0$, so that no event horizon is present when $\text{r}>{r}_{h}$. Also, for $\text{r}={r}_{h}$, the metric function (3) becomes $G\left(\text{r}\right)=0.$ In addition, the metric function can be written in the form: $G\left(\text{r}\right)=\frac{2b}{r} \left(\text{r}-{r}_{h}\right)\left(\text{r}-{r}_{n}\right),$ where ${r}_{n}=-\frac{1}{4b} \left(1+\sqrt{1+16bm}\right),$ ^40^. Thus, from a negative value of ${r}_{n}$, it cannot be explained as the horizon. So, the RMS metric possesses only the event horizon, ${r}_{h}=\frac{1}{4b} \left(-1+\sqrt{1+16bm}\right).$

Moreover, the metric (3) with $(1>9{m}^{2}{\Lambda }_{ext}>0)$ becomes zero at two positive values of $\text{r}$ corresponding to two real positive roots. When ${m}^{2}{\Lambda }_{ext}>\frac{1}{9}$, the metric function becomes negative, so let ${m}^{2}{\Lambda }_{ext}\le \frac{1}{9},$ ^7^.

Thus, if $\left(0<\Lambda {m}^{2}\le \frac{1}{9}\right),$ the metric has two horizons one is the event horizon and the second is the cosmological horizon. For increasing $m$, ${r}_{h}$ and keeping ${\Lambda }_{ext}$ constant, the cosmological horizon will decrease. Therefore, both horizons coincide at $r={r}_{h}= {r}_{c}=3m.$ In most cases, we let ${m}_{-}=1$, so the cosmological horizon becomes dimensionless and equal $\Lambda {m}_{-}^{2}$. Consequently, the radius of the throat is always considered in the region outside the event horizon of BH ($\text{r}>{r}_{h}$). Therefore, for the considered parameter values there is no ${r}_{c}$ but only ${r}_{h}.$

Afterward, the geometry of TS surrounding wormhole spacetime is developed by the cut-and-paste approach.

The single manifold $M$, bounded by hypersurface $\Sigma$ is obtained by gluing together the two regions ${M}^{+}$ and ${M}^{-}$ at their boundaries, $M={M}^{+}\cup {M}^{-},$ with natural identification of the boundaries (hypersurface) $\Sigma \equiv {\Sigma }^{+}={\Sigma }^{-}.$

For the time-like hypersurface $\Sigma \equiv {\Sigma }^{\pm }=\left\{{r}^{\pm }=a|a>{r}_{h}\right\}$. We set the values of $a$ (radius of the throat) greater than the event horizon radius ${r}_{h}$ to avoid the presence of horizons and singularities in the line element (2),^43^.

Assuming that $h_{ + } = h_{ - } = r^{2}$ is at the location of the throat, and the time evolution $a(\tau )$ is described by the throat radius ${r}_{\pm }=a(\tau )$, with the proper time $\tau$ along the hypersurface. Consequently, on the hypersurface $\Sigma ,$ the induced (intrinsic) metric is defined by

4 $$ds^{2} = - d\tau^{2} + h\left( {a\left( \tau \right)} \right)\left( {d\theta^{2} + \sin^{2} \theta d\varphi^{2} } \right).$$

Furthermore, according to the Darmois–Israel formalism introduced by Israel^3^, the extrinsic curvature across the hypersurface Σ, can be expressed as follows,

5 $$K_{ij}^{ \pm } = - n_{\gamma }^{ \pm } \left. {\left( {\frac{{\partial^{2} x^{\gamma } }}{{\partial \xi^{i} \partial \xi^{j} }} + \Gamma_{\alpha \beta }^{\gamma } \frac{{\partial x^{\alpha } }}{{\partial \xi^{i} }}\frac{{\partial x^{\beta } }}{{\partial \xi^{j} }}} \right)} \right|_{\Sigma } ,$$

where $\xi^{i}$ and ${\chi }_{\pm }^{\alpha }$ are the coordinates on Σ and coordinates in ${M}^{\pm }$, while ${\Gamma }_{\alpha \beta }^{\gamma }$ and $n_{\gamma }^{ \pm }$ correspond to Christoffel symbols and the four-unit normal vector.

Moreover, the extrinsic curvatures of the metric (3) are described by

6 $$K_{\theta \theta }^{ \pm } = \pm \frac{{h^{\prime}}}{2}\sqrt {G\left( a \right) + \dot{a}^{2} } \equiv K_{\varphi \varphi }^{ \pm } ,\quad K_{\tau \tau }^{ \pm } = \mp \frac{1}{{G^{2} \sqrt {\dot{a}^{2} + G} }}\left[ { - G^{2} \ddot{a} - \acute{G}^{\prime}\left( {\dot{a}^{4} + G\dot{a}^{2} + \frac{1}{2 } G^{2} } \right)} \right],$$

in which the dot and the prime represent the derivatives of $\tau$ and $a$, respectively.

Accordingly, the surface stress-energy tensor ${S}_{j}^{i}=diag(-\sigma ,{p}_{\theta },{p}_{\varphi })$, related to the discontinuity of ${K}_{ij}^{\pm }$ at Σ, is described by the Lanczos equation^5^,

7 $$S_{ij} = \frac{ - 1}{{8\pi }}\left( {\left[ {K_{ij} } \right] - \left[ K \right]g_{ij} } \right),$$

with $\left[ K \right]$ representing the trace of $\left[ {K_{ij} } \right] = K_{ij}^{ + } - K_{ij}^{ - }$, while $\sigma$ and $p$ represent the surface energy density and pressure, respectively. Therefore, Lanczos equations become

8 $$\sigma = \frac{ - 1}{{4\pi }}\left[ {K_{\theta }^{\theta } } \right],$$

9 $$p = p_{\theta } = p_{\varphi } = \frac{1}{8\pi }\left( { \left[ {K_{\tau }^{\tau } } \right] + \left[ {K_{\theta }^{\theta } } \right] } \right).$$

Consequently, by inserting (6) into (8) and (9) to obtain

10 $$\sigma = \frac{{ - \acute{h}}}{4\pi h}\left( {\sqrt {G_{ + } + \dot{a}^{2} } - \sqrt {G_{ - } + \dot{a}^{2} } } \right),$$

11 $$p = \frac{1}{16\pi }\left( {\frac{{2\ddot{a} + G^{\prime}_{ + } }}{{\sqrt {G_{ + } + \dot{a}^{2} } }} - \frac{{2\ddot{a} + G^{\prime}_{ - } }}{{\sqrt {G_{ - } + \dot{a}^{2} } }}} \right) - \frac{1}{2}\sigma .$$

It is observed that the negative sign of ($\sigma < 0$) in Eq. (10) and the flare-out condition $\stackrel{`}{h}>0,$ indicate that the matter at the throat is exotic. There are several energy conditions applied to the matter of the theory depending on the source of the energy–momentum tensor. It represents a common property to the states of matter and the field. It means that the distribution of matter at the throat violates the weak energy condition ($\sigma \ge 0$, $\sigma + p \ge 0$) because $\sigma$ is negative^44^. The null energy conditions require only that ($\sigma + p \ge 0$), from a static solution of (10) and (11) that $\sigma +p<0$, this condition is violated. While the strong energy condition holds by $\sigma +3p\ge 0$ and $\sigma + p \ge 0$ is satisfied for $\Lambda <0$ and violated for $\Lambda >0$.

The recent cosmological observations, such as the cosmic microwave background radiation, type Ia supernovae (SNIa), and large-scale redshift structure, confirmed that our universe is undergoing a phase of accelerated expansion. The source of this acceleration is described by an exotic type of fluid with negative pressure called dark energy. The simplest two candidates of dark energy (DE) are the universe filled with exotic fluid and the second one is the cosmological constant. One exotic matter is Chaplygin gas (CG) which behaves like a pressureless fluid at an era and a cosmological constant at a later era.

Thus, a universe may be described starting from the radiation stage to the dominated stage by DE consistently. Consequently, the general modification of CG (called MGCG) is suitable to describe the evolution of the universe over a wide range epoch. And also, MGCG is a fluid model which unifies DE and dark matter depending on the suitable choices of involved parameters^34^.

Therefore, to study the stability analysis within the context of RMSdS spacetime, we consider an exotic background fluid called MGCG whose EoS, Debnath et al*.*^45^, is described by

12 $$p = \alpha \sigma - \frac{\gamma }{{\sigma^{\beta } }},$$

where $0<\beta \le 1$, $\alpha <0$, and $\gamma \ge 0$ are free parameters.

When $\alpha =0$ and $\beta =1$ Eq. (12) is reduced to Chaplygin gas and reduced to generalized Chaplygin gas with $\alpha =0$. Also, a cosmological constant emerges by setting $\beta =0$ and $\gamma =1+\alpha$. While, for $\gamma =0$ it reduces to an EoS which describes a phantom energy with $\alpha =-\delta$ and a quintessence fluid with $\alpha =\delta$.

Furthermore, using the Eqs. (10), (11) and (12), the dynamical evolution becomes

13 $$\begin{aligned} & - 2\ddot{a}\left( {\sqrt {\dot{a}^{2} + G_{ + } } - \sqrt {\dot{a}^{2} + G_{ - } } } \right)^{\beta + 1} + \left( {\sqrt {\dot{a}^{2} + G_{ + } } - \sqrt {\dot{a}^{2} + G_{ - } } } \right)^{\beta } \left( {G^{\prime}_{ + } \sqrt {\dot{a}^{2} + G_{ - } } - G^{\prime}_{ - } \sqrt {\dot{a}^{2} + G_{ + } } } \right) \\ & + \frac{{h^{\prime}}}{h}\left( {2\alpha + 1} \right)\left( {\sqrt {\dot{a}^{2} + G_{ + } } - \sqrt {\dot{a}^{2} + G_{ - } } } \right)^{\beta + 1} \sqrt {\dot{a}^{2} + G_{ + } } \sqrt {\dot{a}^{2} + G_{ - } } + 16\pi \gamma ( - 8\pi \frac{h}{{h^{\prime}}})^{\beta } \sqrt {\dot{a}^{2} + G_{ + } } \sqrt {\dot{a}^{2} + G_{ - } } = 0. \\ \end{aligned}$$

Alternatively, the continuity equation is described by

14 $$\frac{d}{d\tau }\left( {h\sigma } \right) + p\frac{d}{d\tau }h = 0,$$

and can be written in the form:

15 $$\sigma^{\prime} = - \frac{{h^{\prime}}}{h}\left( {\sigma + p} \right).$$

Accordingly, using Eqs. (12) and (15) to obtain

16 $$\sigma^{\prime} = - \frac{{h^{\prime}}}{h}\left[ {\sigma \left( {1 + \alpha } \right) - \frac{\gamma }{{\sigma^{\beta } }}} \right].$$

Thus, the second derivative of ${\sigma }^{\prime}$ becomes

17 $$\sigma^{\prime\prime} = \left( {\frac{{h^{\prime}}}{h}} \right)^{2} \left\{ {\left( {\sigma + p} \right)\left[ {\left( {2 + \varepsilon^{2} } \right) - \frac{{hh^{\prime\prime}}}{{h^{\prime 2} }}} \right]} \right\}.$$

As a result, the solution of Eq. (16) is determined by

18 $$\sigma^{\beta + 1} = \frac{\gamma }{{\left( {\alpha + 1} \right)}}\left( {1 - \left( {\frac{{h_{^\circ } }}{h}} \right)^{n} } \right) + \left( {\frac{{h_{^\circ } }}{h}} \right)^{n} \sigma_{ \circ }^{\beta + 1} ,$$

with $$n=(\alpha +1)(\beta +1)$$.

In addition, from Eq. (10) the dynamical equation becomes

19 $$\dot{a}^{2} + H\left( a \right) = 0,$$

in which the effective potential $H(a)$ is described by

20 $$H\left( a \right) = \frac{1}{2}\left( {G_{ + } + G_{ - } } \right) - \frac{{(h^{\prime})^{2} }}{{(16\pi h)^{2} \sigma^{2} }}(G_{ + } - G_{ - } )^{2} - \left( {\frac{4\pi h}{{h^{\prime}}}} \right)^{2} \sigma^{2} .$$

Consequently, a linearization is going to be done to determine whether and in what conditions the static solution with a radius ${a}_{\circ }$ is stable under a linear radial perturbation about ${a}_{\circ }$. To analyze the stability, the Taylor expansion up to the second order at ${a}_{\circ }$ of the effective potential is employed

21 $${\text{H}}\left( {\text{a}} \right) = {\text{H}}\left( {a_{ \circ } } \right) + {\text{H}}^{\prime } \left( {a_{ \circ } } \right)\left( {a - a_{ \circ } } \right) + \frac{1}{2}{\text{H}}^{\prime \prime } \left( {a_{ \circ } } \right)(a - a_{ \circ } )^{2} + O\left[ {(a - a_{ \circ } )^{3} } \right] .$$

Moreover, the first and second derivatives of $\text{H}(a)$ take the following form:

22 $${\text{H}}^{\prime } \left( a \right) = A^{\prime} + \left( {\frac{1}{8\pi W}} \right)^{2} \left[ { - T^{\prime} + W^{\prime}\left( { \frac{2T}{W} - 2\left( {16\pi } \right)^{2} W^{3} } \right)} \right],$$

23 $$H^{\prime\prime}\left( a \right) = A^{\prime\prime} + \frac{1}{{(8\pi W^{2} )^{2} }}\left\{ { - W^{2} T^{\prime\prime}} \right. + 4WW^{\prime}T^{\prime} + 2W^{\prime\prime}[WT - \left( {16\pi^{2} )^{2} W^{5} } \right]\left. { - 2{\text{W}}^{\prime 2} \left[ {3T + \left( {16\pi^{2} W^{2} } \right)^{2} } \right] } \right\},$$

where $$W=\frac{2h}{{h}^{\prime}}\sigma$$, $$A=\frac{1}{2}({G}_{+}+{G}_{-})$$ and $$T=({G}_{+}-{G}_{-}{)}^{2}$$.

Inserting Eqs. (15), (17) and $h={a}^{2}$ into Eq. (23) to obtain:

24 $$\begin{aligned} & H^{\prime\prime}\left( a \right) = A^{\prime\prime} + \frac{1}{{(8\pi W^{2} )^{2} }}\left\{ { - W^{2} T^{\prime\prime}} \right. - 4WT^{\prime}\left( {\sigma + 2p} \right) + \frac{4}{{a^{2} }}\left( {\sigma + p} \right)\left( {3 + 2\varepsilon^{2} } \right) \\ & [WT - \left( {16\pi^{2} )^{2} W^{5} } \right]\left. { - 2\left( {\sigma + 2p} \right)^{2} \left[ {3T + \left( {16\pi^{2} W^{2} } \right)^{2} } \right] } \right\}, \\ \end{aligned}$$

where $\upvarepsilon ^{2} ( = {\text{dp}}/{\text{d}}\upsigma )$ is called the squared speed of sound.

From this perspective, the effective potential is approximated as a linear function around $a={a}_{\circ }$. Thus, the necessary general conditions for the existence and stability of a static fluid shell $\text{H}({a}_{\circ })=0$ and ${\text{H}}^{\prime}({a}_{\circ })=0$ are satisfied. As a result, the dynamical equation can be expressed as ${\dot{a}}^{2}\left(\tau \right)=-\frac{1}{2}{\text{H}}^{^{\prime\prime} }({a}_{\circ })(a-{a}_{\circ }{)}^{2}+O\left[(a-{a}_{\circ }{)}^{3}\right]$. The stability condition is examined by considering a linear perturbation and depends on the sign of ${\text{H}}^{^{\prime\prime} }({a}_{\circ })$ at the throat radius. A stable configuration is achieved when ${\text{H}}^{^{\prime\prime} }({a}_{\circ })>0$, while ${\text{H}}^{^{\prime\prime} }({a}_{\circ })<0$ indicates instability.

Consequently, from Eq. (24), the square speed of sound ${\upvarepsilon }^{2}$ takes the form:

25 $$\begin{aligned} \varepsilon^{2} & = - \frac{1}{2} + \frac{h}{{4h^{\prime} {\text{W}}\left( {\sigma + p} \right){ }[\left( {16\pi^{2} )^{2} W^{4} - T} \right]}}\left\{ {W^{4} A^{\prime\prime}} \right.(8\pi )^{2} - W^{2} T^{\prime\prime} - 4WT^{\prime}\left( {\sigma + 2p} \right) \\ & \left. { - 2\left( {\sigma + 2p} \right)^{2} \left[ {3T + \left( {16\pi^{2} W^{2} } \right)^{2} } \right] } \right\}. \\ \end{aligned}$$

Afterward, it is worth noting that the static solution ($\ddot{a}=\dot{a}=0$) of Eqs. (10) and (11) simplifies to

26 $$\sigma = \frac{{ - h^{\prime}}}{4\pi h}\left( {\sqrt {G_{ + } } - \sqrt {G_{ - } } } \right),$$

27 $$p = \frac{1}{16\pi }\left( {\frac{{G^{\prime}_{ + } }}{{\sqrt {G_{ + } } }} - \frac{{G^{\prime}_{ - } }}{{\sqrt {G_{ - } } }}} \right) - \frac{1}{2}\sigma .$$

Furthermore, the dynamical evolution (13) becomes

28 $$\left( {\sqrt {G_{ + } } - \sqrt {G_{ - } } } \right)^{\beta } \left( {G^{\prime}_{ + } \sqrt {G_{ - } } - G^{\prime}_{ - } \sqrt {G_{ + } } } \right) + \frac{{\left( {2\alpha + 1} \right)h^{\prime} }}{h}\left( {\sqrt {G_{ + } } - \sqrt {G_{ - } } } \right)^{\beta + 1} \sqrt {G_{ + } } \sqrt {G_{ - } } + 16\pi \gamma \left( {\frac{ - 8\pi h}{{h^{\prime}}}} \right)^{\beta } \sqrt {G_{ + } } \sqrt {G_{ - } } = 0.$$

Consequently, in the case of SRdS and Rindler-Schwarzschild (RS) spacetimes, let us assume that: $m_{ - } = m{ ,}$ $m_{ + } = \left( {1 + \omega } \right)m$, $b_{ \pm } = b$, ${\Lambda }_{ - } = 0,$ and ${\Lambda }_{ + } = {\Lambda ;}$ therefore, the metric (3) takes the following form

29 $$G_{ + } = 1 - \frac{{2m_{ + } }}{a} + 2 ba - \frac{1}{3}{\Lambda }a^{2} ,\quad G_{ - } = 1 - \frac{{2m_{ - } }}{a} + 2 ba\quad {\text{and}}\quad h_{ \pm } = a^{2} .$$

Moreover, by plugging the Eq. (29) into (27) and (28), to obtain

30 $$\sigma \left( {a_{ \circ } } \right) = \frac{ - 1}{{2\pi a_{ \circ }^{2} }}\left( {\sqrt {a_{ \circ }^{2} - 2ma_{ \circ } \left( {\omega + 1} \right) + 2ba_{ \circ }^{3} - \frac{1}{3}{\Lambda }a_{ \circ }^{4} } - \sqrt {a_{ \circ }^{2} - 2ma_{ \circ } + 2ba_{ \circ }^{3} } } \right),$$

31 $$p\left( {a_{ \circ } } \right) = \frac{1}{{8\pi a_{ \circ }^{2} }}\left( {\frac{{m\left( {\omega + 1} \right) + 2ba_{ \circ }^{2} - \frac{1}{3}{\Lambda }a_{ \circ }^{2} }}{{\sqrt {a_{ \circ }^{2} - 2ma_{ \circ } \left( {\omega + 1} \right) + 2ba_{ \circ }^{3} - \frac{1}{3}{\Lambda }a_{ \circ }^{4} } }} - \frac{{m + ba_{ \circ }^{2} }}{{\sqrt {a_{ \circ }^{2} - 2ma_{ \circ } + 2ba_{ \circ }^{3} } }}} \right) - \frac{1}{2}{\upsigma }\left( {a_{ \circ } } \right).$$

Also, by inserting (29) into (20) to get

32 $$H\left( a \right) = \frac{1}{{a^{2} }}\left[ {a^{2} - ma\left( {\omega + 2} \right) + 2ba^{3} - \frac{1}{6}{\Lambda }a^{4} } \right] - \frac{{(m\omega )^{2} }}{{(4\pi a^{2} )^{2} \sigma^{2} }} - (2\pi a)^{2} \sigma^{2} .$$

Meanwhile, inserting Eqs. (29), (30) and (31) into (25) to become

33 $$\begin{aligned} \varepsilon^{2} & = - \frac{1}{2} + \frac{1}{{4L Z \left( {J a^{2} - 4m^{2} \omega^{2} } \right)}}\left\{ { - \frac{\pi Ja}{4}\left[ {2m\left( {\omega + 2} \right) + \frac{1}{3}\Lambda a^{3} } \right]} \right. - \frac{{2L^{2} }}{{\pi a^{2} }}\left[ {3m^{2} \omega^{2} - \frac{1}{3}\Lambda {\text{m}}\upomega a^{3} + \frac{1}{9}\Lambda^{2} a^{6} } \right] \\ & \quad + \left. {16 L E \left( {m\omega } \right)\left( {m\omega - \frac{1}{3}\Lambda a^{3} } \right) - 2\uppi a^{2} E^{2} \left( {J a^{2} + 12m^{2} \omega^{2} } \right)} \right\}, \\ \end{aligned}$$

with,

$$\begin{aligned} & L \equiv 2\pi a^{2} \sigma = \left( {\sqrt {a^{2} - 2ma + 2ba^{3} } - \sqrt {a^{2} - 2\left( {\omega + 1} \right)ma + 2ba^{3} - \frac{1}{3}{\Lambda }a^{4} } } \right), \\ & E \equiv \sigma + 2p = \frac{1}{4\pi a}\left( {\frac{{\left( {\omega + 1} \right)m + ba^{2} - \frac{1}{3}{\Lambda }a^{2} }}{{\sqrt {a^{2} - 2\left( {\omega + 1} \right)ma + 2ba^{3} - \frac{1}{3}{\Lambda }a^{4} } }} - \frac{{m + ba^{2} }}{{\sqrt {a^{2} - 2ma + 2ba^{3} } }}} \right), \\ & Z \equiv \sigma + p = \frac{1}{{4\pi a^{2} }}\left( {2\pi a^{2} E + L} \right),\quad J \equiv (16\pi^{2} a^{2} )^{2} \left( {\frac{L}{{2\pi a^{2} }}} \right)^{4} = \frac{{16L^{4} }}{{a^{4} }}. \\ \end{aligned}$$

**Figure 1:**
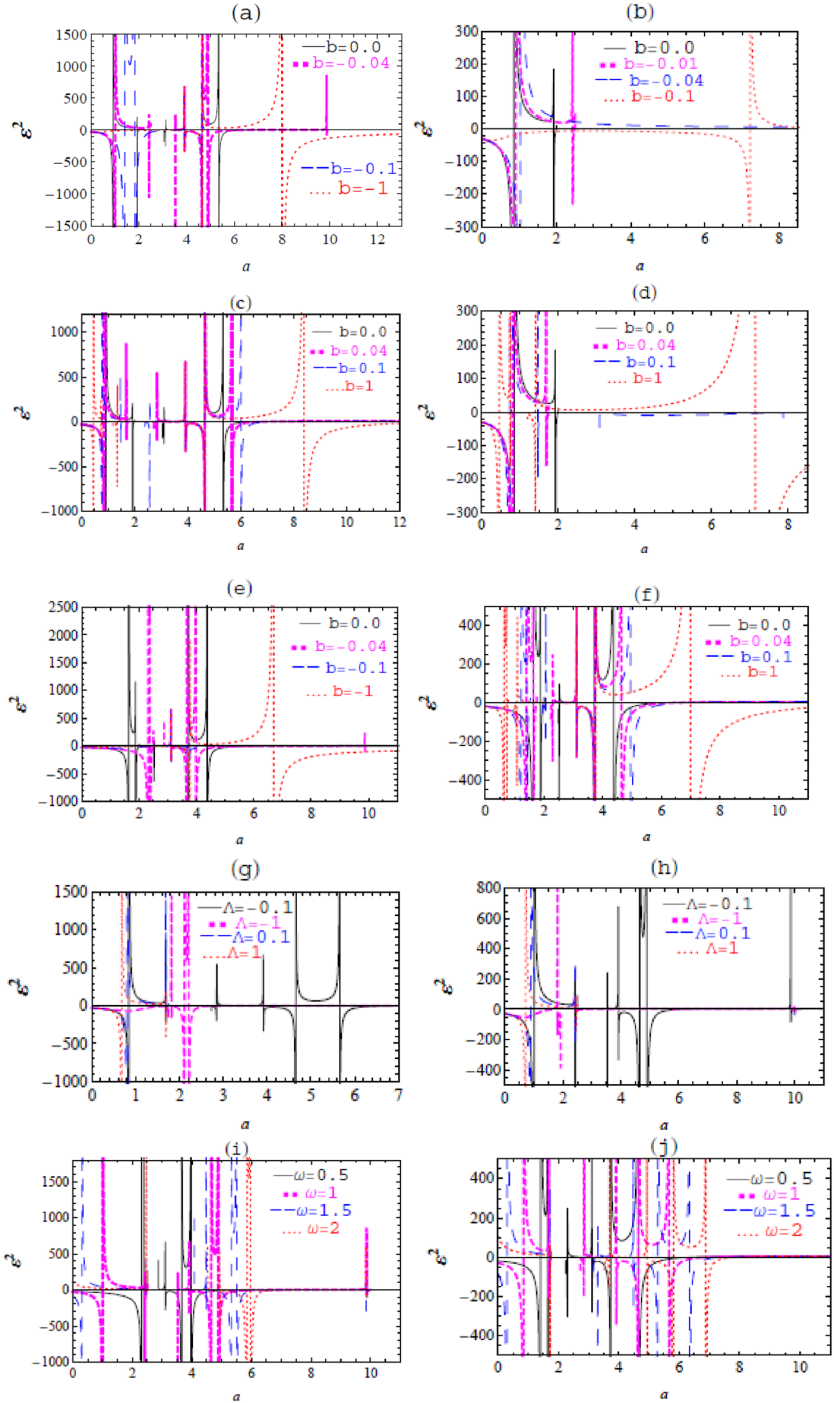
The graph of ${\varepsilon }^{2}$ against $a$ with $m=1$ and different values of: (**a**) $\Lambda =-0.1,\omega =1,$ $b=0, -0.04, -0.1, -1,$ (**b**) $\Lambda =0.1,$ $\omega =1$ $, b=0, -0.04, -0.1, -1,$ (**c**) $\Lambda =-0.1,$ $\omega =1,$ $b=0, 0.04, 0.1, 1,$ (**d**) $\Lambda =0.1,$ $\omega =1,$ $b=0, 0.04, 0.1, 1,$ (**e**) $\Lambda =-0.1,$ $\omega =0.5,$ $b=0, -0.04,- 0.1,- 1,$ (**f**) $\Lambda =-0.1,$ $\omega =0.5,$ $b=0, 0.04, 0.1, 1,$ (**g**) $\Lambda =-0.1,-\text{1,0.1,1},$ $\omega =1,$ $b=0.04,$ (**h**) $\Lambda =-0.1,-\text{1,0.1,1},$ $\omega =1,$ $b=- 0.04,$ (**i**) $\Lambda =-0.1,$ $\omega =\text{0.5,1},\text{1.5,2},$ $b=-0.04,$ (**j**)$\Lambda =-0.1,$ $\omega =\text{0.5,1},\text{1.5,2},$ $b= 0.04.$

The graph of ${\varepsilon }^{2}$ against $a$ is presented in Fig. 1**,** having different values of $m,\omega ,b,$ and $\Lambda$ as free parameters.

**Figure 2:**
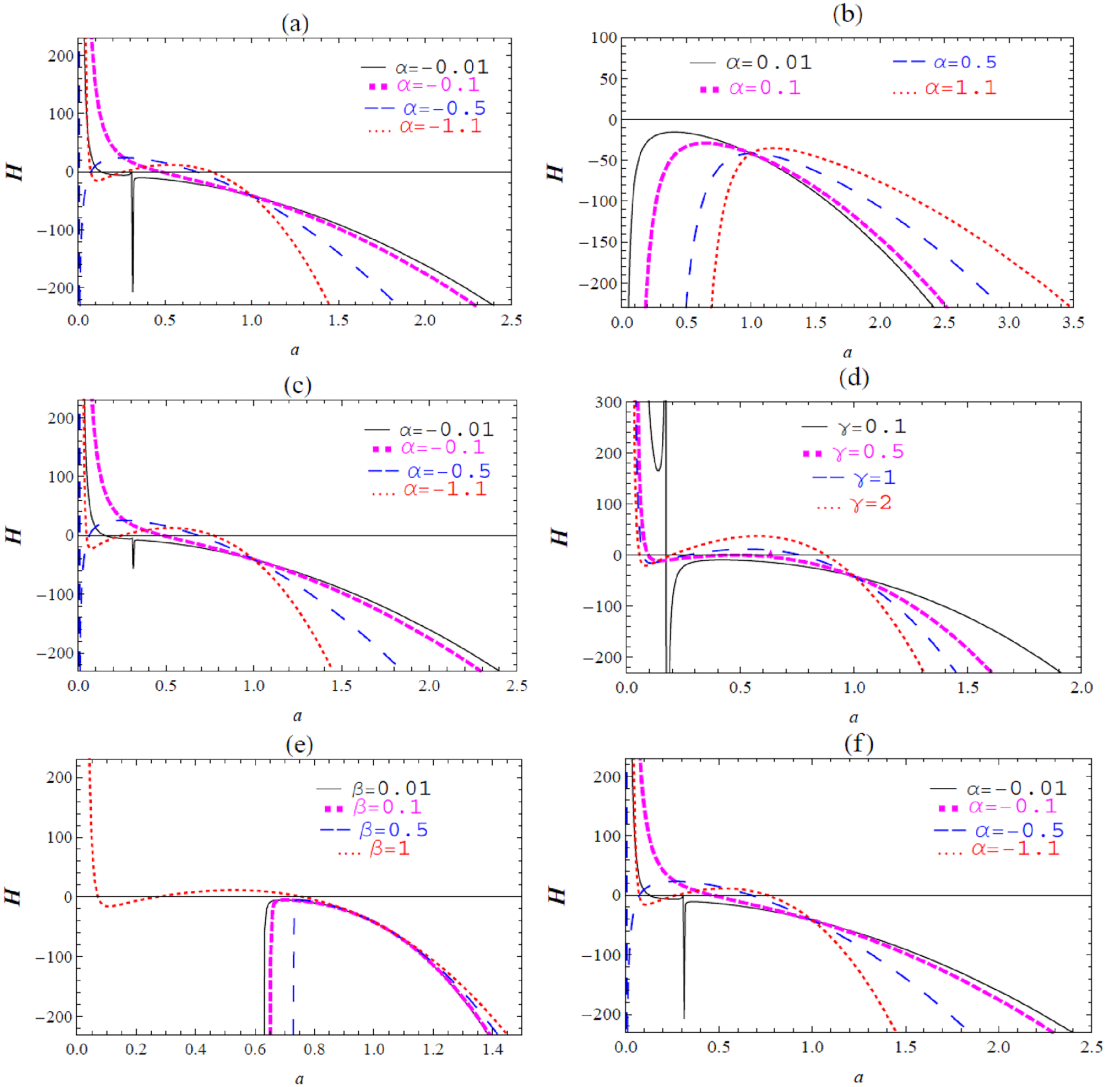
The graph of $\text{H}\left(a\right)$ against $a$ according to $m=1, {a}_{\circ }=1,$ and ${\sigma }_{\circ }=1$ for different values of: (**a**) $\Lambda =1,\omega =1,$ $\gamma =1, \beta =1,b=0.04,$ $\alpha =-0.01,- 0.1, -0.5, -1.1,$ (**b**) $\Lambda =1,\omega =1, \gamma =1, \beta =1, b=0.04,$ $\alpha =0.01, 0.1,$ $0.5, 1.1,$ (**c**) $\Lambda =1,\omega =0.5,$ $\gamma =1, \beta =1,b=0.04,$ $\alpha =-0.01,- 0.1, -0.5, -1.1,$ (**d**) $\Lambda =1,\omega =1, \gamma =\text{0.1,0.5,1},2, \beta =1, b=-0.04,$ $\alpha =- 1.1,$ (**e**) $\Lambda =1,\omega =1,$ $\gamma =1, \beta =\text{0.01,0.1}, \text{0.5,1}, b=-0.04,$ $\alpha = -1.1,$ (**f**) $\Lambda =1,\omega =1, \gamma =1, \beta =1, b=-0.04,$ $\alpha =-0.01,-0.1-0.5,- 1.1.$

The graphical representation of the effective potential $\text{H}\left(a\right)$ against $a$, obtained from Eqs. (32) and (18), is presented in Fig. 2. Various values of $m,{b,a}_{\circ },{\sigma }_{^\circ },\omega ,\alpha ,\gamma ,\beta$ and $\Lambda$ are considered as free parameters.

The graphs of ${\varepsilon }^{2}$ versus $a$ are given in Fig. 1 for different values of $m,\upomega ,\text{b},$ and $\Lambda$ as free parameters. The stability configuration increases due to increasing $\Lambda ,$ in both cases of $\Lambda >0$ and $\Lambda <0$. Also, the stability configuration in the case of $\Lambda <0$ is greater than the stability in the case of $\Lambda >0.$ Similarly, the stability region increases by increasing $\upomega$. Furthermore, the stability region decreases by decreasing the Rindler parameter $\text{b}$ in the case of $b <0,$ while for $\text{b}>0,$ the stable region increases by decreasing b.

Furthermore, the variation between $\text{H}$ and $a$ is given in Fig. 2**.** The stability configurations can be extended by changing the value of the parameter $\alpha$. The graphs are not changed with $\Lambda$ and $b$ parameters. While the stability configurations can be slightly extended due to $\upomega$. Moreover, the graphs exhibit notable changes with changing $\upbeta$ and $\upgamma$ parameters.

**Figure 3:**
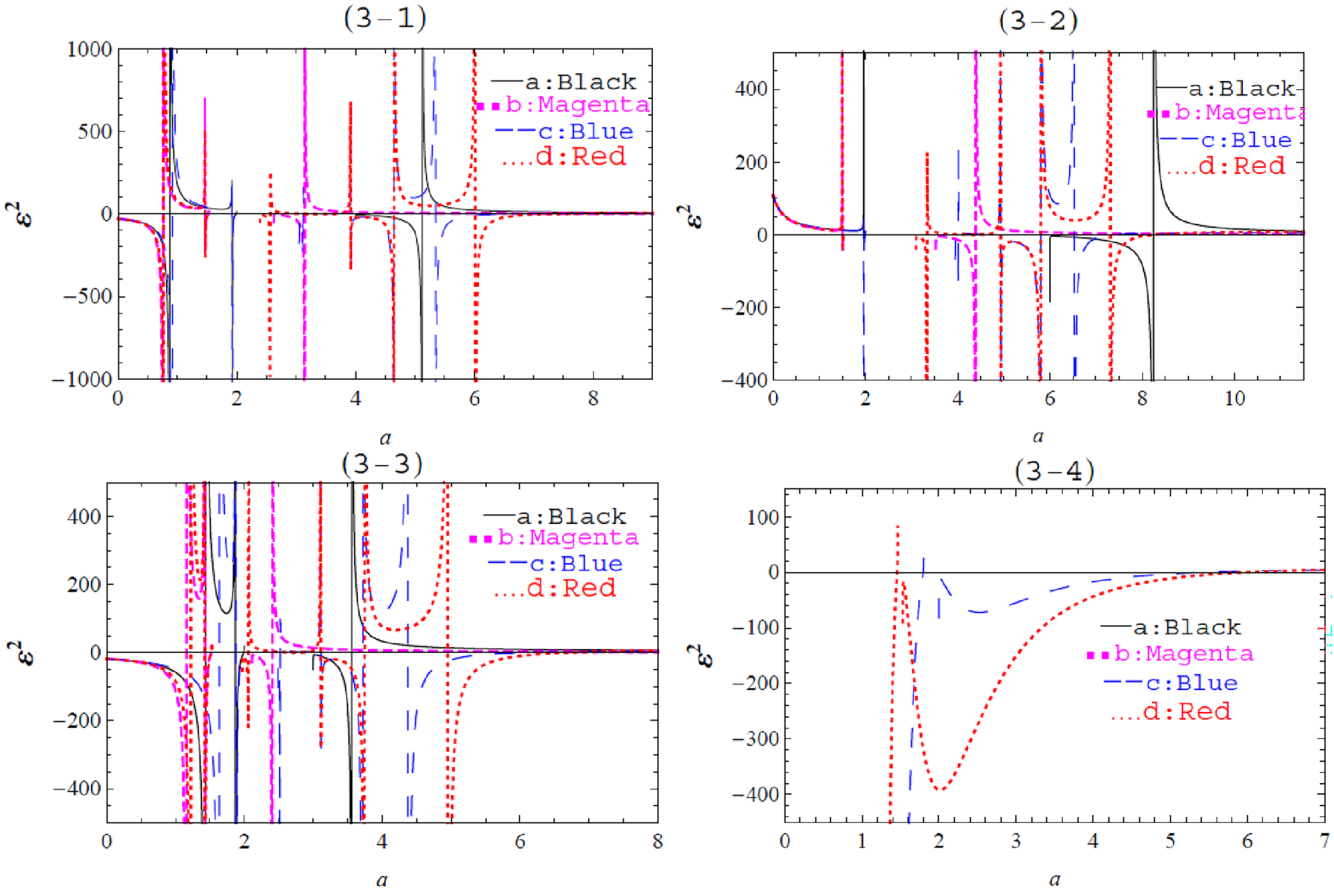
The graph of ${\varepsilon }^{2}$ against $a$ with $m=1$ and different values of $\Lambda ,b,$ and $\omega .$ Fig. 3–1: $\omega =1$ and (**a**) $b=0,$ $\Lambda =0,$ (**b**) $b=0.1,$ $\Lambda =0,$ (**c**) $b=0,$ $\Lambda =-0.1,$ (**d**) $b=0.1,$ $\Lambda =-0.1.$ Fig. 3–2: $\omega =2$ and (**a**) $b=0,$ $\Lambda =0,$ (**b**) $b=0.1,$ $\Lambda =0,$ (**c**) $b=0,$ $\Lambda =-0.1,$ (**d**) $b=0.1,$ $\Lambda =-0.1.$ Fig. 3–3: $\omega =0.5$ and (**a**) $b=0,$ $\Lambda =0,$ (**b**) $b=0.1,$ $\Lambda =0,$ (**c**) $b=0,$ $\Lambda =-0.1,$ (**d**) $b=0.1,$ $\Lambda =-0.1.$ Fig. 3–4: $\omega =0$ and (**a**) $b=0,$ $\Lambda =0,$ (**b**) $b=0.1,$ $\Lambda =0,$ (**c**) $b=0,$ $\Lambda =-0.1,$ (**d**) $b=0.1,$ $\Lambda =-0.1.$

**Figure 4:**
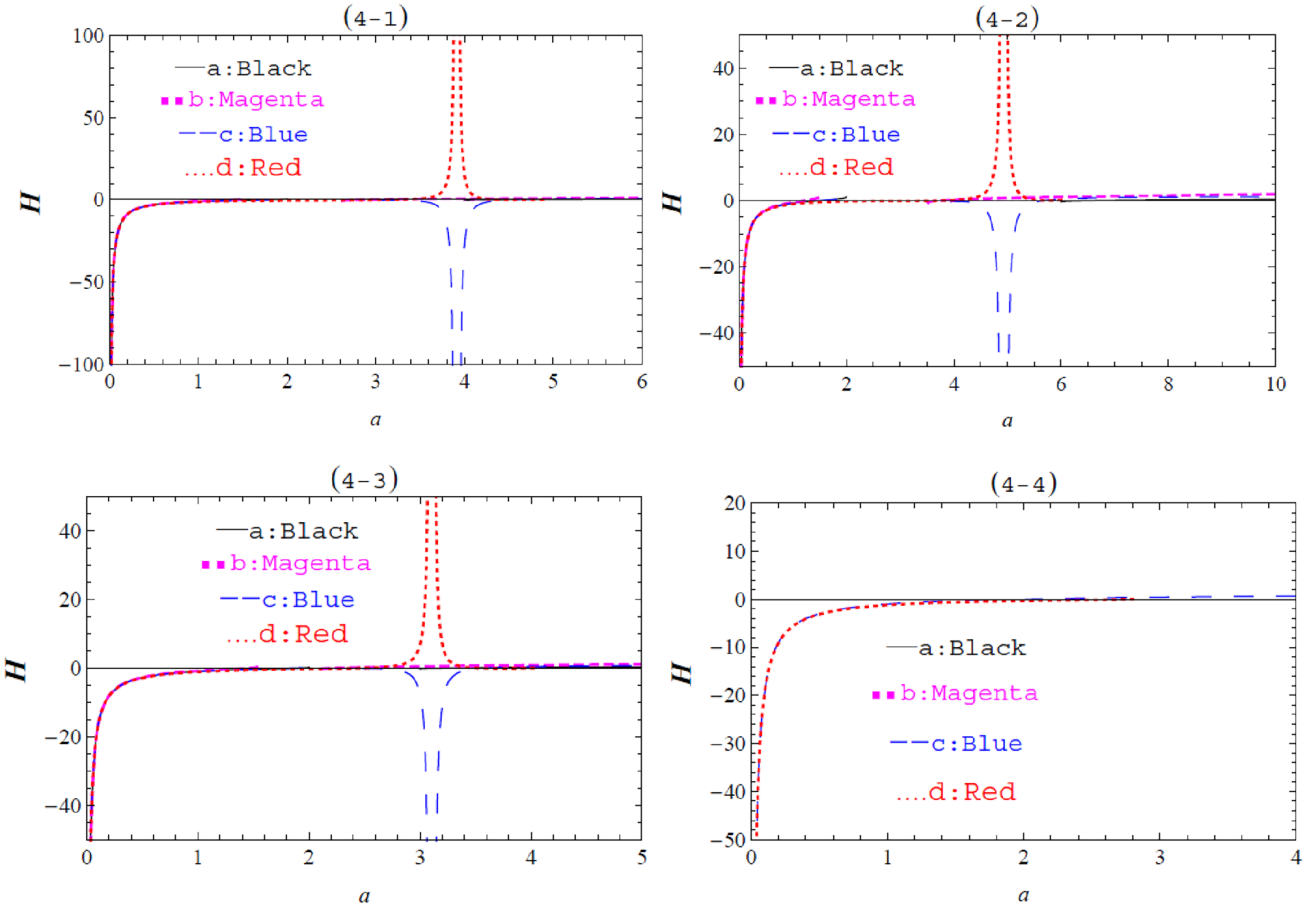
The graph of $\text{H}\left(a\right)$ against $a$ with $m=1$ and different values of $\Lambda ,b,$ and $\omega .$ Fig. 4–1: $\omega =1$ and (**a**) $b=0,$ $\Lambda =0,$ (**b**) $b=0.1,$ $\Lambda =0,$ (**c**) $b=0,$ $\Lambda =-0.1,$ (**d**) $b=-0.1,$ $\Lambda =-0.1.$ Fig. 4–2: $\omega =2$ and (**a**) $b=0,$ $\Lambda =0,$ (**b**) $b=0.1,$ $\Lambda =0,$ (**c**) $b=0,$ $\Lambda =-0.1,$ (**d**) $b=-0.1,$ $\Lambda =-0.1.$ Fig. 4–3: $\omega =0.5$ and (**a**) $b=0,$ $\Lambda =0,$ (**b**) $b=0.1,$ $\Lambda =0,$ (**c**) $b=0,$ $\Lambda =-0.1,$ (**d**) $b=-0.1,$ $\Lambda =-0.1.$ Fig. 4–4: $\omega =0$ and (**a**) $b=0,$ $\Lambda =0,$ (**b**) $b=0.1,$ $\Lambda =0,$ (**c**) $b=0,$ $\Lambda =-0.1,$ (**d**) $b=-0.1,$ $\Lambda =-0.1.$

To obtain a comparison for the stability configuration of the Schwarzschild, Schwarzschild–Rindler, Schwarzschild-de Sitter, and Schwarzschild–Rindler–de Sitter metrics, we graph Figs. 3 and 4, and the results are tabulated in Table 1.

**Table 1:** A comparison of the stability configuration of the proposed models.

Model	Line color in figure	Parameters	Figure 3:${\varepsilon }^{2}(a)$				Figure 4:$\text{H}\left(a\right)$			
			3–1	3–2	3–3	3–4	4–1	4–2	4–3	4–4
Schwarzschild	a: Black	$b=0,$	$\omega =1$	$\omega =2$	$\omega =0.5$	$\omega =0$	$\omega =1$	$\omega =2$	$\omega =0.5$	$\omega =0$
		$\Lambda =0$	$\omega =1$	$\omega =2$	$\omega =0.5$	$\omega =0$	$\omega =1$	$\omega =2$	$\omega =0.5$	$\omega =0$
Schwarzschild–Rindler	b: Magenta	$b=0.1,$	$\omega =1$	$\omega =2$	$\omega =0.5$	$\omega =0$	$\omega =1$	$\omega =2$	$\omega =0.5$	$\omega =0$
		$\Lambda =0$	$\omega =1$	$\omega =2$	$\omega =0.5$	$\omega =0$	$\omega =1$	$\omega =2$	$\omega =0.5$	$\omega =0$
Schwarzschild–de Sitter	c: Blue	$b=0,$	$\omega =1$	$\omega =2$	$\omega =0.5$	$\omega =0$	$\omega =1$	$\omega =2$	$\omega =0.5$	$\omega =0$
		$\Lambda =-0.1$	$\omega =1$	$\omega =2$	$\omega =0.5$	$\omega =0$	$\omega =1$	$\omega =2$	$\omega =0.5$	$\omega =0$
Schwarzschild–Rindler–de Sitter	d: Red	$b=0.1,$	$\omega =1$	$\omega =2$	$\omega =0.5$	$\omega =0$	$\omega =1$	$\omega =2$	$\omega =0.5$	$\omega =0$
		$\Lambda =-0.1$	$\omega =1$	$\omega =2$	$\omega =0.5$	$\omega =0$	$\omega =1$	$\omega =2$	$\omega =0.5$	$\omega =0$

The graph of ${\varepsilon }^{2}$ against $a$ is plotted in Fig. 3**,** with different values of $m,\omega ,b,$ and $\Lambda$ as free parameters.

The graphical representation of the effective potential $\text{H}\left(a\right)$ against $a$, obtained from Eqs. (32) and (30), is plotted in Fig. 4. Various values of $m,\omega ,b,$ and $\Lambda$ are considered free parameters.

The stability of thin shell wormholes for Schwarzschild, Schwarzschild–Rindler, Schwarzschild-de Sitter, and Schwarzschild–Rindler–de Sitter metrics is given in Table 1.

## Conclusion

The study focuses on the dynamics of asymmetric traversable wormholes that connect two distinct spacetimes, namely the RS metric and the SRdS metric. The analysis is conducted using cut-and-paste techniques within the framework of the Darmois-Israel formalism. Additionally, the stability analysis of these asymmetric TSWs is investigated by considering linear radial perturbations and incorporating the modified generalized Chaplygin gas EoS. Furthermore, new results regarding the stability and dynamics of these asymmetric TSWs are presented. It may be that the asymmetric TSW is stable at ${\text{H}}^{^{\prime\prime} }({a}_{\circ })>0$, while at ${\text{H}}^{^{\prime\prime} }\left({a}_{\circ }\right)<0$ it turns out to be unstable. It is observed that the stable configurations depend on the appropriate values of different parameters included in the metric spacetimes and the MGCG EoS. We have found that the stability regions are greatly affected by the presence of the Rindler parameter as well as the cosmological constant. In summary, we concluded that the influence of the Rindler parameter increases the stability configurations, and the stability configurations are also extended by increasing the value of the cosmological constant. Also, the influence of changing the EoS parameters increases the stability regions.

## Data Availability

The data sets used and analyzed during the present study are available from the corresponding author upon reasonable request.
